# Incidence, prevalence and mortality of Parkinson’s disease in Greece

**DOI:** 10.1007/s10072-025-08297-2

**Published:** 2025-07-12

**Authors:** George Makris, Evangelia Samoli, Gregory Chlouverakis, Cleanthe Spanaki

**Affiliations:** 1 Department of Neurology, School of Medicine, University of Crete, Heraklion, 74100 Greece; 2 Department of Hygiene and Epidemiology, School of Medicine, National Kapodistrian University of Athens, Athens, 11527 Greece; 3 Biostatistics Lab, School of Medicine, University of Crete, Heraklion, 74100 Greece

**Keywords:** Epidemiology, Incidence, Mortality, Parkinson’s disease, Prevalence

## Abstract

**Introduction:**

Parkinson’s disease is a progressive neurological condition with significant social burden, expected to rise in the future. Epidemiological research is essential to inform data-driven health policies and unravel new insights into its pathogenesis and potential prevention.

**Methods:**

We used a drug prescription database with extensive population coverage to calculate, nationwide and regional, incidence and prevalence rates of Parkinson’s disease. Additionally, we calculated mortality rates, crude case fatality risk and mortality rate ratios relative to the general population.

**Results:**

The crude incidence, prevalence and mortality rates were estimated at 48 cases per 100,000 person-years [95% CI 47–50], 400 cases per 100,000 persons [95% CI 396–404] and 71 deaths per 100,000 person-years [95% CI 69–72] respectively. A male predominance was observed across all rates within age-groups. The mortality rate ratios for Parkinson disease patients aged 70–79 and > 80 compared to the general population were estimated at 6.6 [95% CI 6.3–6.9] and 2.0 [95% CI 1.9–2.0] respectively. Incidence and prevalence varied significantly by region with the lowest incidence recorded in Crete at 35 cases per 100,000 person-years [95% CI 30–39] and the highest in Thessaly at 69 cases per 100,000 person-years [95% CI 63–76].

**Conclusions:**

Our results provide robust epidemiological evidence to guide the strategic allocation of health system and social resources for Parkinson’s disease in Greece. Additionally, they set the stage for further analytical epidemiological studies to elucidate factors contributing to geographical variations of the disease burden.

**Supplementary Information:**

The online version contains supplementary material available at 10.1007/s10072-025-08297-2.

## Introduction

Parkinson’s disease is an age-related, progressive neurodegenerative disorder characterized both by motor and non-motor symptoms. While it is the second most common neurodegenerative disease globally, recent evidence suggests that it shows the fastest-growing prevalence and associated disability among neurodegenerative conditions [1, 2]. Unfortunately, only symptomatic treatments are currently offered to the patients as disease modifying or preventative treatments are not available yet. Considering the globally aging population and the increasing burden of Parkinson’s disease for patients, carers, families and health care systems, epidemiological research is of paramount importance for optimal health and social resource allocation decisions. Additionally, data on Parkinson’s disease epidemiology can be instrumental in unraveling important environmental and genetic factors associated with its pathogenesis that may be operational in specific geographical areas.

Global estimates of the epidemiological burden of Parkinson’s disease rely on imperfect data [3], while high-quality country specific epidemiological studies are only available in resource-rich settings. In Greece, only two cohort studies have provided estimates– one for incidence and the other for prevalence. Incidence was estimated at 41 cases per 100,000 person-years based on 88 cases in 25,407 participants nationwide that were followed-up for 214,505 person-years [4]. However, case ascertainment in that study was mainly based on self-reported data, while the geographical representativeness of the data was not reported. Prevalence of Parkinson disease in Greece was estimated among 1,765 volunteers over 65 years of age in two cities (Larissa and Athens) at 1,9% [5]. This estimate, however, has low precision due to the small number of identified cases (*n* = 34), and restricted geographical coverage. These limitations indicate a significant gap in the availability of valid and representative descriptive epidemiological data of Parkinson’s disease in Greece.

Collecting high-quality epidemiological information, with a focus on spatial and social representativeness is one of the key recommendations proposed by the WHO, under the aegis of the global intersectoral plan for neurological disorders [6]. In line with the global effort to better appreciate the burden of Parkinson’s disease and its known geographic variability worldwide, the aim of this study is to provide a comprehensive estimate of the disease’s epidemiological burden in Greece. We hope that our analysis will contribute essential data to assess Parkinson’s disease socioeconomic impact and to inform future health research and policy decisions.

## Methods

### Data sources

The National Organization for the Provision of Health Services,” EOPYY”, is the major statutory health insurance provider in Greece, managing a single unified health insurance fund that covers over 95% of the population. Prescription medications are reimbursed only when issued through the national electronic prescription platform (“e-prescription”) [7]. The database for electronic prescriptions which records patient demographics, clinical diagnoses and medication details including type and dosages, is developed and operated by the Electronic Social Security Governance company “IDIKA S.A”. This prescription platform is fully integrated with EOPPY and healthcare providers. All electronic drug prescriptions are required to be linked to an ICD-10 (International Classification of Diseases) diagnostic code. Currently, EOPPY reimburses 90% of the cost of medications for Parkinson’s disease.

Following a formal request to the Ministry of Health (MoH), we obtained a dataset from IDIKA S.A. containing all prescriptions linked to Parkinson’s disease (designated by ICD-10 code G20), issued between July 1, 2017, to January 1, 2025. However, until the end of 2019, national electronic prescription protocols specific to Parkinson’s disease had not been established and the incorporation of population groups into EOPPY was still incomplete, introducing potential misclassification bias. Therefore, our analysis focuses on prescriptions issued from January 1, 2020, onward.

Population characteristics were collected from the 2021 census and the mortality data, that are annually compiled, were retrieved from the website of the Greek Statistical Authority [8].

### Case, outcome and location ascertainment

We analyzed Parkinson’s disease related prescriptions (designated by ICD-10 code G20) issued between January 1, 2020, to January 1, 2025. We excluded prescriptions, for which the medications were not dispensed. We included all patients that received antiparkinsonian medications listed as disease-specific treatments in the national therapeutic protocols for Parkinson’s disease [9]. These included levodopa-containing medications, dopamine agonists, monoamine oxidase B inhibitors, anticholinergic medications and amantadine. Additionally, eligible patients were required to have at least three prescription fills within 12 months and to have taken at least one of these medications for longer than 6 months. This criterion was adopted to maximize specificity for Parkinson’s disease. Other causes of parkinsonism do not typically exhibit a sustained beneficial response to levodopa; thus, short treatment trials are often used during the differential diagnosis’ process of PD from non-neurodegenerative parkinsonian syndromes.

Cases of Parkinson’s disease were defined as patients fulfilling the aforementioned criteria. Prevalence within a specific population subgroup was calculated as the mean annual number of Parkinson’s disease cases divided by the number of individuals in that population subgroup. New cases of Parkinson’s disease were defined as patients that had not appeared in any previous year of our dataset. Incidence in a specific population subgroup was calculated as the mean annual new cases of Parkinson’s disease divided by the total number of individuals in that population subgroup. All deaths among patients diagnosed with Parkinson’s disease, were included in mortality calculations, regardless of the recorded direct cause of death. Participants were attributed to geographic regions based on the location of the pharmacy most frequently visited to fill their prescriptions.

### Statistical analysis

We calculated age- and sex- specific as well as overall mean annual numbers of Parkinson’s disease cases, mean annual new cases and related deaths nationwide. Using the 2021 Census data, we estimated age-, sex- and region-specific prevalence, incidence and mortality rates (per 100.000 persons and 100.000 person-years respectively). New cases and incidence were not calculated for 2024, due to the inclusion criteria that require a minimum treatment duration for case ascertainment. Crude case fatality risk (CFR) for Parkinson’s disease was calculated by dividing the mean annual number of deaths among patients by the mean annual number of diagnosed cases over the study period. For mortality rate ratios (MRRs) estimates, we divided age- and sex-specific mortality rates among Parkinson’s disease patients by the corresponding rates in the general population. Given that deaths among individuals with Parkinson’s disease represent a very small proportion of total deaths recorded, we did not subtract them from the denominator in the MRR calculation, as estimates would not be meaningfully affected by their exclusion.

Confidence intervals (CIs) are estimated to account for random sampling error. While, in this study, no sampling error is expected due to the near entire population coverage, we opted to present confidence intervals to account for chronological sampling fluctuations and facilitate comparisons between population subgroups. Confidence intervals were calculated under the assumption of the Poisson distribution. The formula used for the calculation of CIs, includes a natural logarithm transformation and the application of the Delta method [10].All statistical analyses were performed in R software version 4.3.3. Regional maps for PD incidence and prevalence were created in ArcGIS desktop: Release 10 software, using ECDC colour palettes [11].

## Results

Between January 1, 2020, and January 1, 2025, 70,248 unique patients met our study criteria of Parkinson’s disease. A total of 1,808,328 antiparkinsonian prescriptions were dispensed to them during the study period. A total of 23,096 new cases and 37,065 patient deaths were recorded. The mean annual number of Parkinson’s disease cases was 41,912, with an average of 5,073 new cases being diagnosed each year.

### Parkinson’s disease incidence and prevalence in greece, including age-group and sex differences

Incidence and prevalence rates of PD, both overall and stratified by sex and age group are shown in Table 1. The overall incidence is 48 cases per 100,000 person-years [95% CI 47–50], while the overall prevalence is 400 cases per 100,000 persons [95% CI 396–400]. Exponentially rising incidence and prevalence rates are observed in predefined age groups: <40, 40–49, 50–59, 60–69, 70–79, > 80. There is a male predominance regarding incidence and prevalence of Parkinson’s disease within all age groups. In a variation of Simpson’s paradox, the widening gender population gap from the age group 50 onward -where women increasingly outnumber men, particularly after 70 years of age- results in comparable overall incidence rates between genders: 48 cases per 100,000 person-years for men [95%CI 46–50 ] and 49 cases per 100,000 person-years for women [95% CI 47–50]. However, this demographic shift leads to a significantly higher overall prevalence in women (423 cases per 100,000 persons [95% CI 418–429]) compared to men (375 cases per 100,000 persons [95% CI 370–381].

**Table 1:** Incidence and prevalence rates of Parkinson’s disease in greece. Ιncidence reported for years 2020–2023 and prevalence for years 2020–2024

	Incidence per 100.000 person-years [95% CI]			Prevalence per 100.000 persons [95% CI]		
	Overall	Male	Female	Overall	Male	Female
< 40	0 [0–0]	0 [0–1]	0 [0–0]	1 [1–2]	2 [1–2]	1 [0–1]
40–49	3 [2–4]	4 [3–5]	2 [1–3]	16 [14–18]	21 [18–24]	11 [9–13]
50–59	13 [11–15]	15 [13–18]	11 [9–13]	83 [79–88]	96 [89–103]	70 [65–77]
60–69	55 [51–59]	63 [57–70]	48 [43–53]	353 [343–363]	399 [384–415]	311 [298–324]
70–79	189 [180–197]	204 [191–217]	176 [165–187]	1370 [1348–1394]	1462 [1427–1498]	1293 [1263–1324]
> 80	289 [278–302]	326 [307–347]	265 [250–280]	2873 [2835– 2911]	2993 [2932–3055]	2793 [2746–2842]
Overall	48 [47–50]	48 [46–50]	49 [47–50]	400 [396–404]	375 [370–381]	423 [418–429]

### Parkinson’s disease mortality, case fatality risk and mortality rate ratio

The crude mortality rate for persons living with Parkinson’s disease is 71 deaths per 100.000 person-years [95% CI 69–72]. Mortality rates increased progressively with advancing age, peaking at 598 deaths per 100,000 person-years [95% CI 581–615] for people over 80 years of age. While there is a male predominance regarding mortality of patients with Parkinson’s disease across all age groups, overall mortality rates are comparable between genders due to Simpson’s paradox, as explained above. The overall crude annual CFR for persons with Parkinson’s disease is 18% [95% CI 17–18] and while crude CFRs in age groups less than 60 years of age approximate 5%, CFR increases after 60 years of age, peaking at 21% for people over 80 years of age. The MMR, that compares mortality among persons with Parkinson’s disease to the general population, shows an increased crude mortality risk of 14.2 [95% CI 13.9–14. 5] for persons with Parkinson’s disease in comparison to the general population, which is more pronounced in younger age groups and for women. ΜΜRs for age groups that include the majority of patients are 6,6 [95% CI 6.3–6.9] for 70–79 years of age and 2,0 [95% CI 1.9–2] for over 80 years of age. Mortality rates, CFRs and MMRs overall and stratified by age group and sex are presented in Table 2 and Supplemental Fig. 1.

**Table 2:** Mortality rate of persons with Parkinson’s disease, annual case fatality risk of persons with Parkinson’s disease and mortality rate ratio of persons with Parkinson’s disease to the general population from 2020 to 2024 in Greece

	Mortality per 100.000 person-Years [95% CI]			Annual Case Fatality Risk (%) [95% CI]			Mortality Rate Ratio to General Population [95% CI]		
	Overall	Male	Female	Overall	Male	Female	Overall	Male	Female
< 40	0 [0–0]	0 [0–0]	0 [0–0]	5 [1–17]	5 [1–22]	5 [1–45]	112.9 [31.8–399.9]	81.0 [17.2–381.6]	190.8 [21.3–1707.4]
40–49	1 [0–1]	1 [0–2]	0 [0–1]	4 [2–8]	5 [2–9]	4 [2–12]	28.8 [15.9–51.9]	21.7 [10.6–44.7]	42.5 [15.1–119.4]
50–59	4 [4–6]	5 [4–7]	4 [3–5]	5 [4–7]	6 [4–8]	5 [4–8]	12.9 [10,2–16,3]	9.6 [7.1–13.1]	19.2 [13.3–27.6]
60–69	36 [33–40]	45 [40–51]	28 [25–32]	10 [ 9–11]	11 [10–13]	9 [8–10]	10 [9.3–11.1]	8.0 [7.1–9.0]	14.1 [12.3–16.2]
70–79	228 [219–237]	258 [244–273]	202 [191–215]	17 [16–17]	18 [17–19]	16 [15–17]	6.6 [6.3–6.9]	5.4 [5.1–5.7]	8.4 [7.9–8.9]
> 80	598 [581–615]	655 [ 627–684]	560 [539–582]	21 [20–21]	22 [21–23]	20 [19–21]	2.0 [1.9–2.0]	1.9 [1.8–2.0]	2.0 [1.9–2.1]
Overall	71 [69–72]	68 [66–71]	73 [71–75]	18 [17–18]	18 [18–19]	17 [17–18]	14.2 [13.9–14.5]	14.1 [13.7–14.6]	14.3 [13.8–14.7]

### Regional differences in Parkinson’s disease prevalence and incidence

The highest regional prevalence rates were observed in Thessaly, the Ionian islands and Western Greece with 529 cases per 100,000 persons [95% CI 512–546], 477 cases per 100,000 persons [ 95% CI 448–508] and 467 per 100,000 persons [95% CI 451–484] respectively. The lowest regional prevalence rates were observed in the Southern Aegean region and Crete with 282 cases per 100,000 persons [95% CI 264–301] and 311 cases per 100,000 persons [ 95% CI 298–325] respectively.

**Fig. 1:**
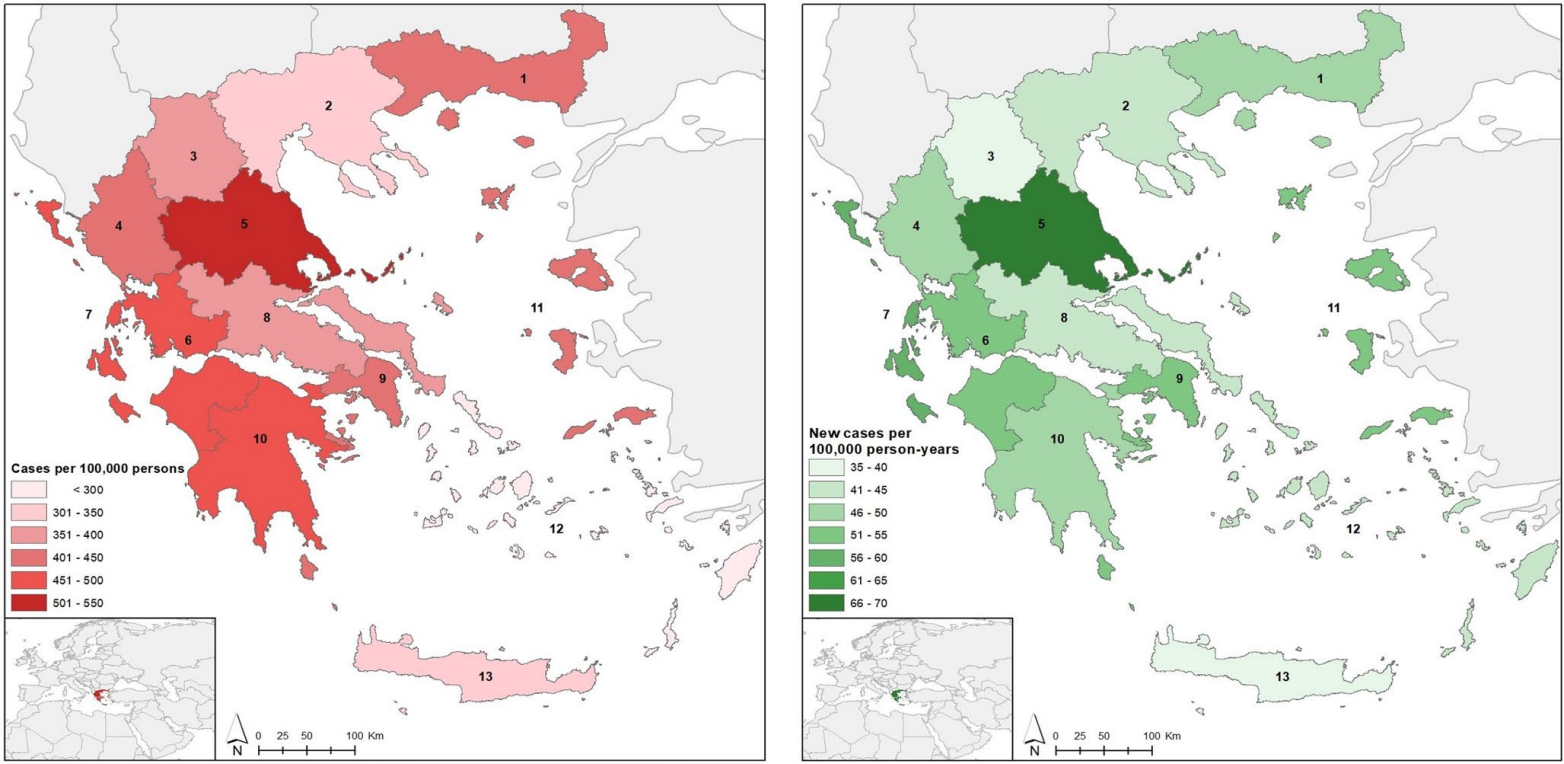
Regional prevalence (left) and incidence(right) rates of Parkinson’s disease in Greece. Ιncidence reported for years 2020–2023 and prevalence for years 2020–2024. (1. Eastern Macedonia and Thrace, 2. Central Macedonia, 3. Western Macedonia, 4. Epirus, 5. Thessaly, 6. Western Greece, 7. Ionian islands, 8. Central Greece, 9. Attica, 10. Peloponnese, 11. Northern Aegean, 12. Southern Aegean, 13. Crete)

The highest incidence rates are found in Thessaly with 69 cases per 100,000 person-years [63–76] and the Ionian islands with 60 cases per 100,000 person-years [50–71]. The lowest incidence rates can be found in Crete with 35 cases per 100,000 person-years [95% CI 30–39], Western Macedonia with 39 per 100,000 person-years [95% CI 32–48] and Southern Aegean with 40 per 100,000 person-years [95% CI 33–47] (shown in Fig. 1). The highest regional mortality rates were observed in Thessaly and the Ionian islands with 83 deaths per 100,000 person-years [95% CI 76–90] and 83 deaths per 100,000 person-years [95% CI 71–96] respectively. The lowest regional mortality rates were observed in in the Southern Aegean region and Crete with 41 deaths per 100,000 person-years [95% CI 35–49] and 55 deaths per 100,000 person-years [ 95% CI 50–61] respectively.

Detailed information on the regional mean annual cases, mean annual new cases, prevalence, incidence and mortality rates is presented in Supplemental Table 1.

## Discussion

### Main findings

In this study, we report descriptive epidemiological measures of Parkinson’s Disease in Greece, using a prescription database thought to include nearly all treated Parkinson’s Disease patients in the country. To our knowledge, this is the first epidemiological study of its kind in Greece.

Our results regarding the crude PD incidence rate of 48 cases [95% CI 47–50] per 100,000 person-years and male predominance within age groups, are in accordance with international findings. A 2016 meta-analysis on the incidence of Parkinson’s Disease that included mostly studies from Europe, North America and Asia reports incidence rates of 37.55 [26.20–53.83, 95% CI] and 61.21 [43.57–85.99, 95% CI] cases per 100.000 person years at risk, for females and males over 40 years of age respectively [12]. There is a well-established male predominance in disease incidence, which is better supported by evidence in the 60–79 age group [12]. More recent large scale observational studies offered estimates for people over 45 years of age ranging from 57 to 140 cases per 100,000 person-years at risk in the UK, depending on the case definition used, and ranging from 47 to 77 per 100,000 person-years at risk in North America [13, 14].

While incidence estimates are available from a few high-resource settings, prevalence data, drawn from a wide range of regions, vary considerably across geography and over time [15]. Prevalence is affected both by incidence and disease duration, which in turn reflects demographics, disease severity, competing health risks and access to and quality of health care. The global all-age prevalence of Parkinson’s disease is reportedly increasing and has reached its highest recorded estimates in the years 2010–2023 at 381 cases per 100,000 persons (95% CI, 267–514 per 100,000), compared to 118 cases per 100,000 persons (95% CI, 77–167 per 100,000) in the years 2000–2009 [16]. Our all-age prevalence estimate of 400 per 100,000 (95% CI, 399–400 per 100,000) is in line with these findings. In contrast to our results, the Global Burden of Disease (GBD) study generated an increasing, albeit significantly lower, all-age prevalence estimate of 152 cases per 100,000 persons [130–181 per 100,000] for 2021 and a projection of 216 cases per 100,000 persons [168–281 per 100,000] for 2050 [1, 17]. However, on the one hand, data reported in this study are limited by the scarcity of reliable epidemiological data in many countries and on the other hand, a lower global crude prevalence rate is expected due to the young and expanding age structure of the global south.

Although Parkinson’s Disease is rarely the direct cause of death, it is associated with an increased risk in mortality. This elevated risk is relevant across all stages of the disease and can be mediated by a variety of pathologies, including infectious and respiratory diseases, cardiovascular complications and external causes, such as falls, accidents related to motor impairment and other violent deaths [14, 18–21]. Similar to prevalence, mortality estimates are heterogeneous across various geographic regions and time periods [15]. Our findings indicate that, like incidence and prevalence, the population mortality burden for patients with Parkinson’s disease is overwhelmingly concentrated among patients over 70 years of age. Nonetheless, the diagnosis of Parkinson’s Disease appears to contribute significantly to mortality in relation to the general population, particularly among women. This relative excess mortality– quantified by the MRR- tends to diminish with increasing age. The most likely explanation for this observation is the presence or absence of competing health risks within different demographic age- or gender-specific subgroups. Possibly, in groups with lower baseline risks, such as younger age groups and women in Greece, the relative impact of Parkinson’s disease becomes more pronounced.

We consider the age-stratified CFRs and MRRs more informative for interpretation than the overall figures. The demographic structure of the general population may inflate the overall MRR, due to the inclusion of larger proportions of younger individuals with low background mortality in the denominator. On the other hand, age-stratified CFRs and MRRs for younger age groups - particularly under 50 years of age- should also be interpreted with caution, as the small numbers of deaths in patients and the general population can contribute to statistical instability and may exaggerate the observed mortality due to rare events.

It is important to note that our crude CFR and MRR estimates, do not reflect the risk of death from the time of disease onset or diagnosis, for which, survival analysis would be appropriate. Instead, these estimates represent the observed, cross-sectional probability of death among individuals living with Parkinson’s disease in specific demographic groups during the study period.

Interestingly, crude mortality in patients with Parkinson’s disease is higher than crude incidence in Greece between 2020 and 2024, indicating a possibly declining prevalence over time. While the reason for this finding remains to be elucidated, longer-term observational studies in Greece are needed to confirm and explain this phenomenon, taking also into account the impact of the SARS-COV-2 pandemic during this period. Despite established projections of increasing prevalence rates for Parkinson’s disease in most settings, there are still large differences to be explained, with some countries in Europe even reporting decreasing rates over time [15].

We also identified significant and concordant regional variations in incidence prevalence and mortality rates of Parkinson’s Disease. For example, Thessaly and the Southern Aegean and Crete regions sit at opposite ends of the incidence and prevalence spectrum indicating that, possible spatial, environmental or genetic, factors could play a role in the pathogenesis or diagnosis of Parkinson’s disease. Previous ecological research in Greece suggests that exposure to blue and green spaces and proximity to the sea, may have protective effects against nervous system diseases [22, 23]. An observational study in Italy has also reported specific risk factors, such as environmental exposure to pesticides, oils and metals, as well as family history of Parkinson’s disease that could also be relevant to the geographic heterogeneity observed in our study [24]. Larger-scale geospatial studies specific to Parkinson’s disease incorporating environmental, behavioral and genetic risk factors in Greece are needed to elucidate our findings.

### Strengths and limitations

#### Validity of prescription studies for PD

While using administrative diagnostic codes can overestimate incidence, there is evidence that using prescription data increases the positive predictive value for the identification of true Parkinson’s disease cases [28, 29]. While large-epidemiological studies and disease registries are vital epidemiological tools, there is added value in using prescription databases, as the former can be labor-intensive, logistically challenging and time-consuming. Given the lack of disease registries in Greece for most non-communicable diseases, including Parkinson’s disease, our findings represent the most comprehensive estimates of disease burden so far.

Additionally, Parkinson’s disease is a privileged condition for this type of research, as diagnosis is almost always synonymous with chronic treatment with medications rendering the possibility of diagnosed patients not receiving antiparkinsonian medications, especially as these are almost completely reimbursed, to be very low.

#### Representativeness

The greatest strength of our study lies in the use of a comprehensive countrywide drug-prescription database, which enables linkage of diagnostic codes, prescription and death records for virtually the entire medication-using population. This ensures that our results are highly representative of the target population and offer robust epidemiological insights.

Geographic representativeness was ensured by using the location of the pharmacy most frequently visited to fill antiparkinsonian prescriptions. This approach overcomes the common limitations of static residential addresses, as usually recorded in administrative databases. This dynamic definition of location may yield more accurate results for health system planning and is well-suited to generate epidemiologic hypotheses. It aligns more closely with models of disease causation that consider long-term environmental exposures, relevant to the field of neurodegenerative diseases. Residual uncertainty regarding patients’ location in the years preceding our analysis period, during which relevant exposures could have occurred, remains a relative limitation for subsequent analytic epidemiological investigation.

#### Case ascertainment and misclassification

Misclassification of Parkinson’s disease can arise both from overdiagnosis– misidentifying other non-degenerative or atypical parkinsonian syndromes as Parkinson’s disease- and underdiagnosis, where symptoms of Parkinson’s disease are attributed to normal aging or other conditions [15]. Our inclusion criteria are designed to prevent misclassification of non-degenerative secondary parkinsonism, which is not levodopa responsive. However, our criteria might not perform as effectively for other neurodegenerative atypical “Parkinson-Plus” syndromes that could have been misdiagnosed as Parkinson’s disease and can partially respond to levodopa treatment for more than 6 months. Due to the rarity of the atypical “Parkinson-plus” syndromes, the proportion of these misdiagnosed cases is unlikely to significantly influence our results [25]. On the other hand, the percentage of undiagnosed Parkinson’s disease increases with age, rising from 18% in individuals aged 65–70 years to 36% for those aged 80–85 years [26]. The net effect of this misclassification bias is difficult to predict across different study settings and populations, including ours.

Finally, our epidemiological results may be influenced by migration patterns. While both incoming and outgoing migration to and from Greece could potentially affect our incidence estimates, we expect this effect to be negligible due to the relatively low average age of both immigrant and emigrant populations [27].

### Policy, practice and research implications

In this study, we report population-level estimates of the burden of Parkinson’s disease in Greece, highlighting significant differences between genders, age groups and regions. Quantifying the burden of Parkinson’s disease and its geographic variations is essential to plan health care services, advocate for coverage of insurance costs and ensure availability of newer treatment modalities, as well as increase social awareness. For example, the need for neurologists, movement disorder clinics and specialists, rehabilitation and nutrition professionals, as well as social support can be assessed at the regional level based on the measured burden of disease.

Regarding clinical practice, an important but often overlooked implication of reporting prevalence rates is their utility in evidence-based differential diagnosis via Bayes’ theorem [30]. In this context, the age- and sex-specific prevalence of different parkinsonian syndromes serve as the prior probabilities for differential diagnosis, which can then be refined with distinguishing clinical features and diagnostic testing.

Last, but not least, a key outcome of our study is its potential to guide further epidemiological and clinical research. For instance, the geospatial study of environmental exposures and their association with disease burden may offer new insights into the disease’s complex pathogenesis. Ultimately, such research could support the development of primary and secondary prevention strategies for neurodegeneration.

## Electronic supplementary material

Below is the link to the electronic supplementary material.

Supplementary Material 1
