# Supplementary material for: Incidence, prevalence and mortality of Parkinson’s disease in Greece
Source: Neurol Sci. 2025 Jul 12;46(10):5027–34. doi: 10.1007/s10072-025-08297-2 (PMC12488759; doi:10.1007/s10072-025-08297-2)
Supplement: Supplementary file 1 — Supplementary Material 1 [file 10072_2025_8297_MOESM1_ESM.docx]

| Administrative Region | Mean Annual Cases | Mean Annual New Cases | Prevalence  (per 100.000 persons) [95% CI] | Incidence  (per 100.000 person-years) [95% CI] | Mortality  (per 100.000 person-  years) [95% CI] |
| --- | --- | --- | --- | --- | --- |
| Eastern Macedonia and Thrace | 2320 | 274 | 413 [396 - 430] | 49 [43 - 55] | 71 [64 - 78] |
| Central Macedonia | 6209 | 710 | 346 [337 – 354] | 40 [37 – 43] | 66 [62 – 70] |
| Western Macedonia | 908 | 100 | 356 [334 – 380] | 39 [32 – 48] | 57 [48 – 67] |
| Epirus | 1309 | 155 | 409 [387 – 432] | 48 [41 – 57] | 61 [53 - 70] |
| Thessaly | 3640 | 478 | 529 [512 – 546] | 69 [63 – 76] | 83 [76 – 90] |
| Western Greece | 3029 | 334 | 467 [451 – 484] | 51 [46 – 57] | 79 [72 – 86] |
| Ionian Islands | 976 | 122 | 477 [448 – 508] | 60 [50 – 71] | 83 [71 – 96] |
| Central Greece | 2006 | 220 | 395 [378 – 412] | 43 [38 – 49] | 74 [67 – 82] |
| Attica | 15396 | 1980 | 404 [397 – 410] | 52 [50 – 54] | 74 [71 – 76] |
| Peloponnese | 2440 | 254 | 452 [435 – 471] | 47 [42 – 53] | 80 [73 – 88] |
| Northern Aegean | 811 | 102 | 416 [388 – 446] | 52 [43 – 64] | 72 [61 – 85] |
| Southern Aegean | 925 | 130 | 282 [264 – 301] | 40 [33 – 47] | 41 [35 – 49] |
| Crete | 1943 | 216 | 311 [298 – 325] | 35 [30 – 39] | 55 [50 – 61] |
| Greece | **41912** | **5073** | **400 [396 – 404]** | **48 [47 – 50]** | **71 [69 – 72]** |

Supplemental Table 1. Mean annual cases, mean annual new cases, overall prevalence, incidence and mortality rates per region in Greece. Incidence reported for years 2020-2023 and prevalence and mortality for years 2020-2024


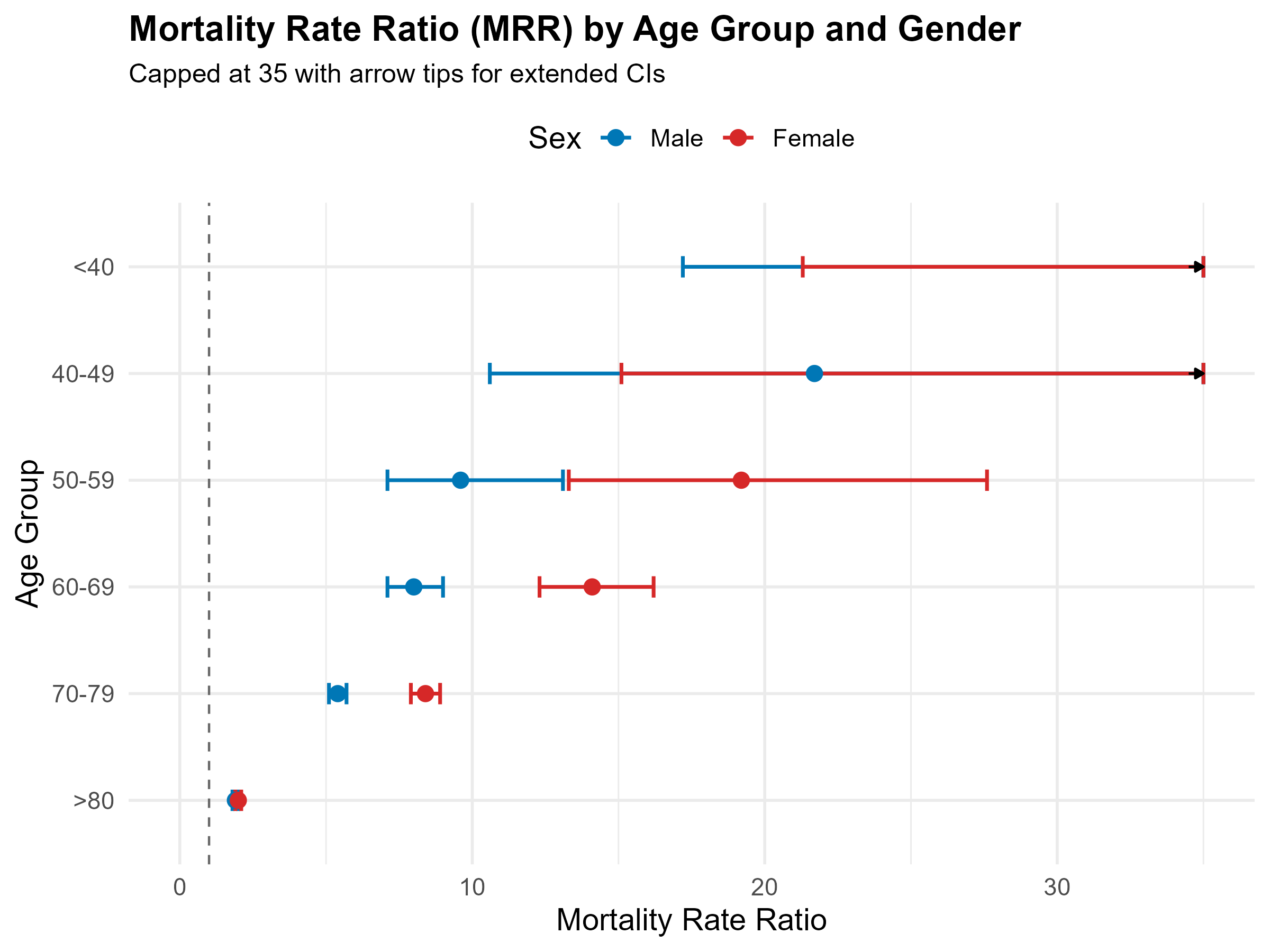

Supplemental Figure 1. Sex- and Age- stratified Mortality Rate Ratios of patients with Parkinson’s Disease compared to the general population (2020-2024)
